# Avian Magnetoreception: Elaborate Iron Mineral Containing Dendrites in the Upper Beak Seem to Be a Common Feature of Birds

**DOI:** 10.1371/journal.pone.0009231

**Published:** 2010-02-16

**Authors:** Gerald Falkenberg, Gerta Fleissner, Kirsten Schuchardt, Markus Kuehbacher, Peter Thalau, Henrik Mouritsen, Dominik Heyers, Gerd Wellenreuther, Guenther Fleissner

**Affiliations:** 1 Hamburger Synchrotronstrahlungslabor HASYLAB at Deutsches Elektronen-Synchrotron (DESY), Hamburg, Germany; 2 Institut für Zellbiologie und Neurowissenschaften, Goethe-Universität, Frankfurt a. M., Germany; 3 Abt. Elementanalytik, Helmholtz Centre Berlin for Materials and Energy, Berlin, Germany; 4 Institut für Biologie und Umweltwissenschaften, Carl von Ossietzky-Universität, Oldenburg, Germany; Lund University, Sweden

## Abstract

The magnetic field sensors enabling birds to extract orientational information from the Earth's magnetic field have remained enigmatic. Our previously published results from homing pigeons have made us suggest that the iron containing sensory dendrites in the inner dermal lining of the upper beak are a candidate structure for such an avian magnetometer system. Here we show that similar structures occur in two species of migratory birds (garden warbler, *Sylvia borin* and European robin, *Erithacus rubecula*) and a non-migratory bird, the domestic chicken (*Gallus gallus*). In all these bird species, histological data have revealed dendrites of similar shape and size, all containing iron minerals within distinct subcellular compartments of nervous terminals of the median branch of the Nervus ophthalmicus. We also used microscopic X-ray absorption spectroscopy analyses to identify the involved iron minerals to be almost completely Fe III-oxides. Magnetite (Fe II/III) may also occur in these structures, but not as a major Fe constituent. Our data suggest that this complex dendritic system in the beak is a common feature of birds, and that it may form an essential sensory basis for the evolution of at least certain types of magnetic field guided behavior.

## Introduction

Unlike most plants, which are fixed to the substrate in which they grow, animals are generally motile. They must move through their daily and seasonally changing environment in order to find, for example, food, sexual partners or nest sites. For this free moving life style, animals must have evolved navigational systems that enable them to identify where they are, how to reach a distant goal, and how to return to a previously visited location. Many environmental clues including visual, olfactory, magnetic and mechanical references have been shown to play a role during orientation in space. Specialized sensory systems combined with complex perception mechanisms have been proposed to evaluate, process and remember these stimuli for navigation.

During the last decades growing evidence has shown that magnetoreception is a relevant mechanism for the orientation and navigation [1]–[7] as well as in other behavioral or physiological categories of many different animal phyla [8]–[10]. The magnetic sense must therefore be addressed as an additional and stand-alone sense, besides vision, hearing, olfaction, taste, electroreception and mechanosensation.

Experimental studies of magnetic field guided behavior have focused on migratory animals like birds (for review: [6], reptiles [11] or fish [12]), but evidence also exists from experiments with mammals (mole rat [13]; bat [14]) and several invertebrate species (crustaceans [15]; honey bees [16]; cockroaches [17]; marine molluscs [18]). Despite the overwhelming behavioral evidence, the molecular, physiological, and cognitive mechanisms enabling animals to sense and extract useful information from the geomagnetic field remain obscure.

Currently, two biophysical mechanisms have emerged as the most promising magneto-detection candidates, namely iron-mineral-based magnetoreception [19], [20] and chemical (photoreceptor-based) magnetoreception [4]–[6], [21]–[22]. Here we concentrate on the iron-mineral based magnetoreception.

Evidence for an iron-mineral based magnetoreceptor localized in the trigeminal system includes for example: (1) Physiological recordings from the trigeminal nerve of fish (ROS = Ramus ophthalmicus superfacialis [23], [24]) and birds (ROM = Ramus ophthalmicus medialis [25]–[27]), suggesting that magnetic information is transmitted via this nerve. (2) Food location by means of magnetic anomalies [10] and (3) a magnetic “fixed-axis” orientation in case of a disrupted photopigment-based compass [28], [29] make use of magnetic field parameters. (4) Lesions of the ophthalmic branch of the trigeminal nerve prevented conditioned pigeons [30] and fish [31] from detecting a strong magnetic anomaly. (5) An elaborate candidate structure for a magnetoreceptor has been described in dendrites of the dermal lining of the upper beak of homing pigeons by methods of histology [19], [20], [32] and physical characterization [20], [33]–[35]. (6) Theoretical considerations confirm that the complex configuration of ferrimagnetic iron-oxides inside subcellular compartments of the dendrites fulfills the criteria of a magnetoreceptor [19], [36].

The first aim of the present paper is to use histological techniques in order to check whether the delicate structure of iron-containing dendrites in the upper beak [20], [32] is a specialization of homing pigeons or whether it occurs in several systematically widely separated taxa of birds with very different lifestyles, including extreme residents (here represented by domestic chickens), trained homers (here represented by homing pigeons), short to middle distance migrants (here represented by European robins), and long-distance migrants (here represented by garden warblers). If these diverse birds would all have the candidate magnetoreception structure, it would be most likely (1) that it is common to all birds, and (2) that they are using this structure.

The second aim of the current paper is to use high-precision X-ray analyses to explore the composition of iron-minerals within the candidate magnetoreception structure in all four bird species. A similar iron-mineral composition of the candidate magnetoreceptor structure in the beaks of different bird species would support the idea that a general concept for magnetoreception exists, which could help to identify yet unknown magnetosensitive structures in other organisms.

## Materials and Methods

Light microscopic histological studies were performed with isolated beaks from the following species: homing pigeon (*Columba livia*, N = 30), European robin (*Erithacus rubecula*, N = 6), garden warbler (*Sylvia borin*, N = 12) and domestic chicken (*Gallus gallus domesticus*, breed *“White Leghorn”*, N = 10). The experimental birds have been kept in the animal houses of the Zoological Institutes in Frankfurt/Main and Oldenburg Universities. All specimens have participated in behavioral experiments prior to the histological investigations. In all cases, the participating laboratories followed the institutional guidelines for using these animals in research. All animal procedures were approved by the local and national authorities for the use of animals in research according to the disclosure requirements given in §4/§17 TierSchG. (Reg. Praes. Darmstadt, Hessen, Germany). An ethics statement is not required for this project.

### Histology

**Figure 1:**
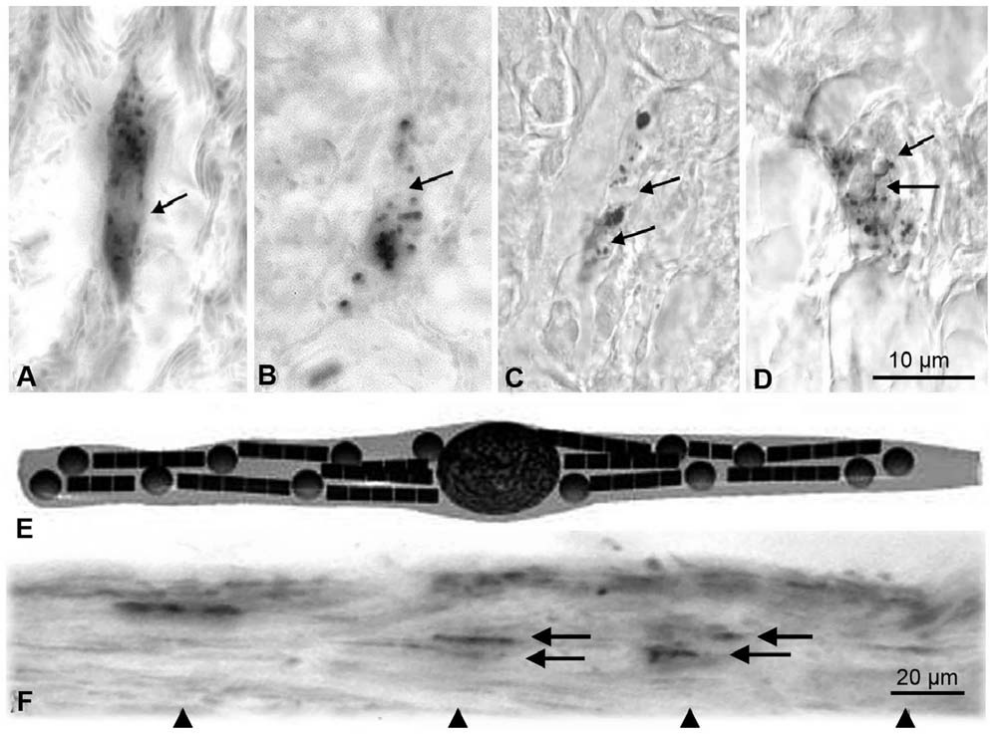
Prussian blue stained dendrites in the inner lining of the upper beak of various bird species. (**A–D**) The dendrites have a similar light microscopic structure irrespective of the avian species (**A** homing pigeon, **B** garden warbler, **C** European robin, **D** domestic chicken). Over a length of about 20 to 30 µm several iron rich bullets (diameter 1 µm) can be found together with a centrally located vesicle (diameter about 5 µm, **arrows** point to vesicles). (Same scale in **A–D**; 10 µm paraffin sections, Prussian blue staining). (**E**) General semi-schematic drawing of an iron containing dendrite (after [19]). (**F**) Axon bundle with several iron containing dendrites. The dendrites are aligned in a distinct micro architecture: Parallel dendrites lie closely attached to each other (arrows). The dendritic groups keep a longitudinal regular distance (arrowheads). (Scale bar 20 µm; sagittal 10 µm thick paraffin section of a pigeon beak; stack reconstruction of different focal planes).

**Figure 2:**
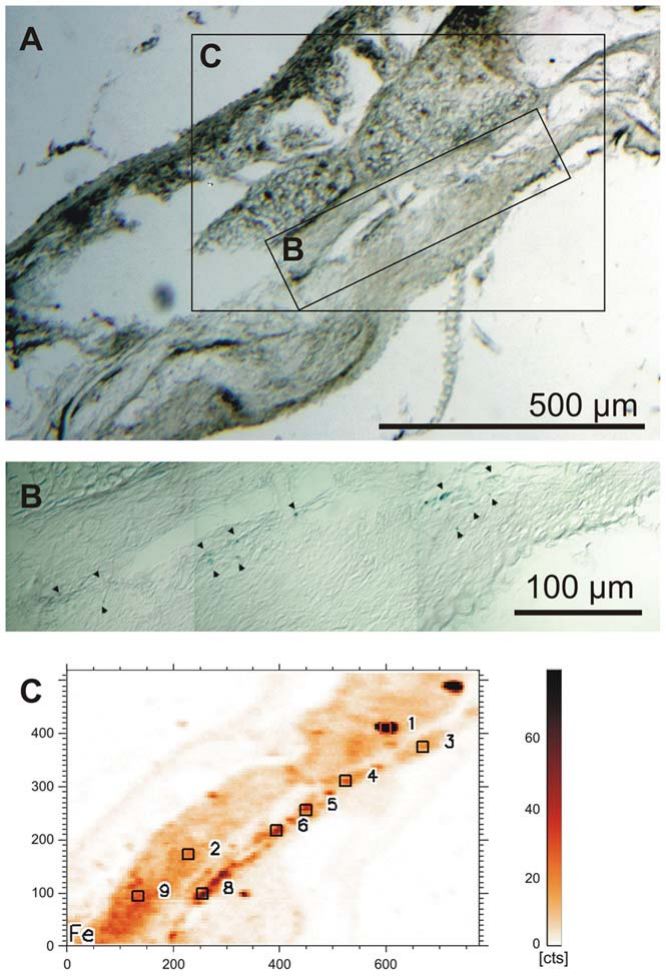
Identification of iron containing dendrites in stained and unstained neighboring sections of a garden warbler beak. (**A**) A Prussian Blue (PB) stained section of the beak of a garden warbler serves as reference. (Scale bar 500 µm; 10 µm thick paraffin section). (**B**) Chain of dendrites identified by Prussian blue staining reconstructed from different focal planes. The major part of these dendrites lies in the unstained neighboring section and was there detected by SXRF (arrow heads point to the dendrites; same section as in **A** at a higher magnification, see frame **B** in **A**; scale bar 100 µm; 10 µm Paraffin section). (**C**) Microscopic SXRF map of Fe of an unstained section neighboring the PB-stained section in **A** (see frame **C** in **A**; section mounted on Ultralene foil). The element map of iron shows a typical accumulation of iron at sites matching PB stained dendrites in **A/B**. (Squares with numbers indicate the measuring points for the subsequent µ-XANES analyses; same scale as in **A**).

For histological processing, the birds received a lethal anesthesia by a high-dosage Narcoren injection followed by transcardial perfusion with 4% Paraformaldehyde. The handling, procedures and chemicals used have been described in detail for homing pigeons [20], [32]. The dermal lining of the upper beak was isolated by means of iron-free Titanium and ceramic tools. Of utmost importance for finding the structural features (see Figure 1, Figure 2A) and a subsequent reliable physical characterization by X-ray analysis are two details of the histological protocol: (1) Perfusion fixation is essential in order to avoid a false positive Prussian Blue (PB)-reaction, e.g., from the iron in the hemoglobin of blood corpuscles, which might be misinterpreted as iron containing magnetosensitive structures. If no perfusion is performed, erythrocytes clotted in capillaries may occur in the X-ray-measurements of unstained parallel sections, and be mistaken for evidence. (2) As iron oxides of small size are readily dissolved in weak acids, it is essential that buffered neutral aqueous solutions are selected and appropriate chemicals for fixation and stabilization are used to keep the iron mineral structures in their natural state: below pH 6.8 the nano/micro-sized iron oxide particles may start to dissolve, above pH 7.6 the Prussian Blue (PB) staining starts fading. The disregard of these prerequisites might be one of the reasons for the delayed discovery of magnetoreceptor candidate structures.

Histological sections were documented by a digital camera (Visitron systems, Puchheim, Germany) attached to a Polyvar microscope (Reichert, Vienna, Austria).

### X-Ray Analysis

**Figure 3:**
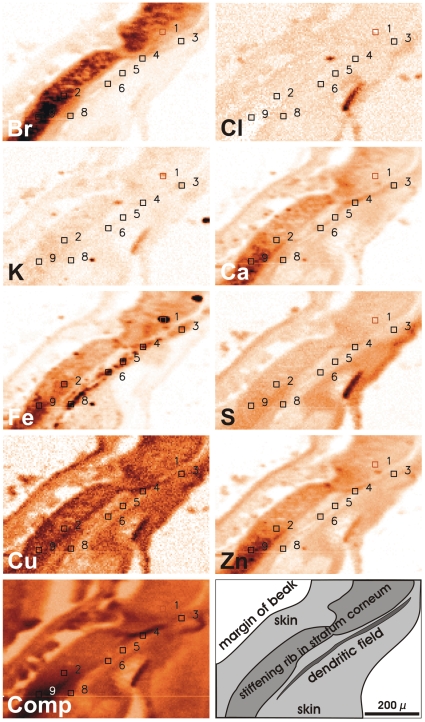
Qualitative distribution of various elements determined by µ-XRF analysis in an unstained section of a warbler beak. According to tissue specificity (see schematic drawing lower right corner), element maps show characteristic differences, which help to further differentiate between the various iron containing structures. For example, dendritic Fe occurs clearly aligned along a delicate structure, an axon bundle, with Ca in a higher concentration compared to the direct vicinity. (The symbols with numbers correspond to the XANES spectra shown in Fig. 5)

**Figure 4:**
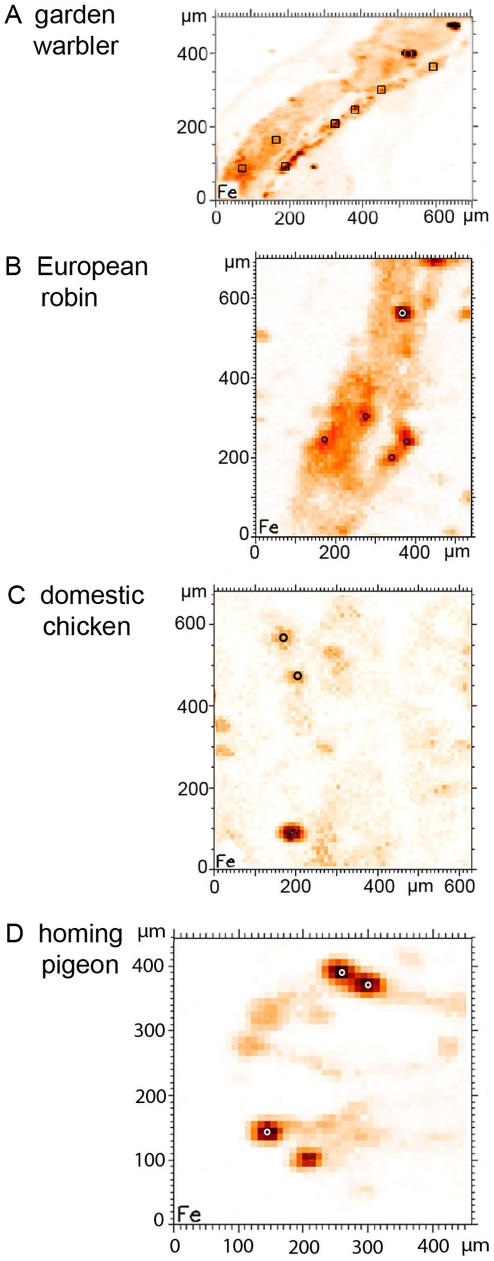
Micro-SXRF Fe-maps of dendritic areas in four avian species. According to the criteria described by Figure 2, areas with iron containing dendrites are selected for micro-SXRF maps in different bird species (**A**) garden warbler, (**B**) European robin, (**C**) domestic chicken, (**D**) homing pigeon. Here the Fe-maps are shown, only. (10 µm sections mounted on Ultralene foil). The measuring sites in these examples of beak sections for the subsequent micro-XANES analyses (see Figure 6) are marked.

All physicochemical studies were performed at the synchrotron hard X-ray microprobe Beamline L at HASYLAB (DESY, Hamburg). Here PB-stained sections served as controls, only (see Figure 2A), while unstained sections neighboring the PB-stained ones - mounted on Ultralene foil (SPEX Certiprep Inc.) - were used for the physical characterization (see Figure 2). Microscopic synchrotron X-ray fluorescence analysis (micro-SXRF) [37] was performed in small areas of the skin which had been pre-selected in the PB stained control sections (see Figure 2, Figure 3, Figure 4). The continuous spectrum of the bending magnet was monochromatized at 17.5 keV by a double multilayer monochromator (NiC). The broad-band monochromatic X-ray beam was focused with polycapillary half-lenses. Two different capillaries were used providing a beam size of 15 µm and 6 µm at a flux of 10^11^ photons/s and 10^10^ photons/s, respectively. The samples were mounted on a XYZ-sample stage in ambient conditions and scanned relative to the X-ray micro-beam in a continuous scanning mode. In the present study, sample times of 0.5-2 s per point were used. Investigated areas and the precision of the measurement were controlled by a long-distance light microscope in transmitted light mode connected with a CCD camera for controlling the areas of interest. Fluorescence photons and scattered radiation were detected by a Silicon drift detector (Vortex EX90, SII Nanotechnology USA Inc.). SXRF-scanning results in the distribution of elements independent of their chemical state (see Figure 2, Figure 3, Figure 4). Elemental maps show the distribution of P, S, Cl, K, Ca, Ti, Mn, Fe, Ni, Cu, Zn, Se, Br, Sr, Ba, Hg, and Pb can be directly correlated with the light-microscopy images of the Prussian Blue-stained neighboring section.

**Figure 5:**
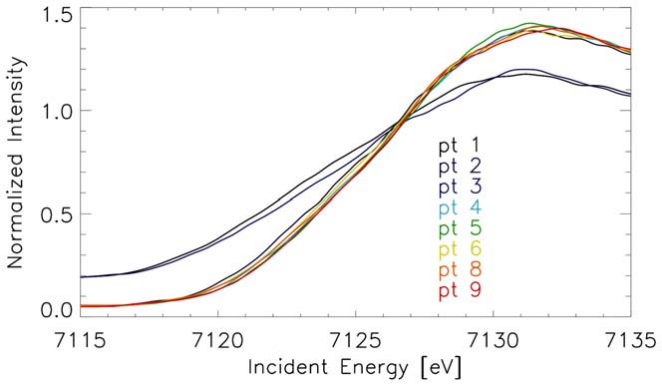
µ-XANES measurements at different sites in an unstained section of the beak of a garden warbler. The spectra measured at the putative dendritic sites (see Figure 3: pt 2, pt 4–9) are very similar, while those of the contamination (pt 1, pt 3) are clearly different and shifted to lower energies. Small deviations of the spectra must be attributed to statistics. (Pt = measuring site)

**Figure 6:**
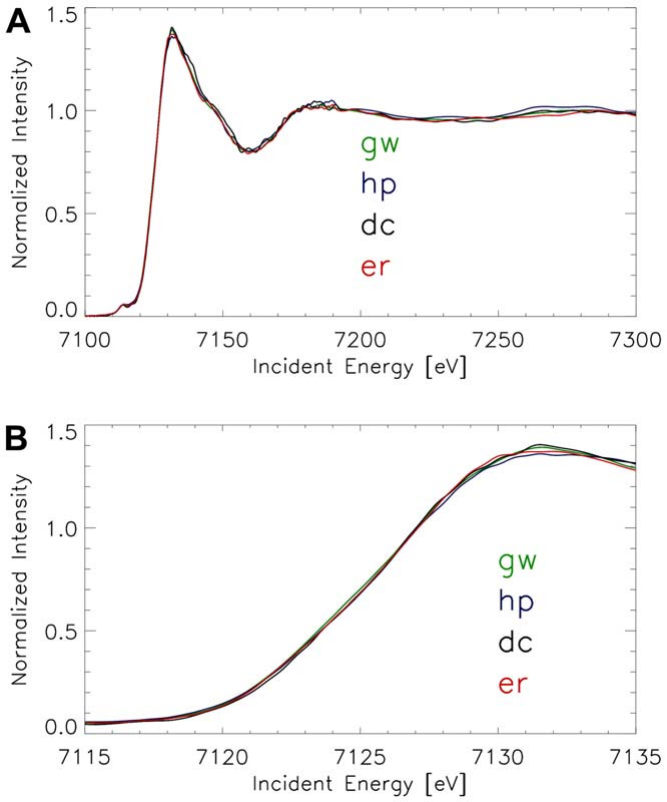
Micro-XANES-spectra of avian dendrites. X-ray spectra at the K-edge of iron of different birds (normalized). The spectra are extremely similar over the complete XANES and extended XANES range. (**A**) XANES-spectra in the energy range between 7100 eV and 7300 eV incident energy, (**B**) The same spectra shown over the energy range between 7115 eV and 7135 eV. All studied avian tissues have spectra of the same edge position and shape. (Averaged and normalized data from several measurements; **green line** = Garden warbler, **blue line** = Homing pigeon, **black line** = Domestic chicken, **red line** = European robin.)

Micro-XANES (microscopic X-ray absorption near-edge structure) spectra were measured at the position of the highest Fe-concentration matching a site inside the prospective sensory dendrite (Figure 5, Figure 6) for the determination of the stoichiometric composition the iron minerals inside the dendrites, especially in order to recognize their putative magnetic features [38]. For spectroscopy, the multilayer monochromator was replaced by a Si (111) monochromator under remote control. A 20 µm diameter X-ray beam of some 10^9^ photons/s was formed by a polycapillary, the way as for the broad-band monochromatic micro-SXRF setup. Before starting the micro-XANES measurement the location of the dendrite was confirmed by a short-range micro-SXRF map. The absorption spectra were recorded in fluorescent mode, tuning the excitation energy near the X-ray absorption K-edge of iron (7112 eV) by stepping the Si (111) monochromator. The fluorescence yield was detected at an angle of 90° to the incoming beam using the Si drift detector. Energy step sizes were chosen as follows: 2 eV from 7050 eV to 7090 eV; 1 eV from 7090 eV to 7105 eV; 0.5 eV from 7105 eV to 7140 eV; 1 eV from 7140 eV to 7200 eV; 5 eV from 7200 eV to 7500 eV. The measuring time for each point was 10 s, and the measurements were repeated several times in order to improve counting statistics. The edge jump of each single measurement was about 1000 counts due to the small amount of Fe in the probed volume (in the picogram range). The absorption signal of a Fe metal foil placed behind the sample was used for a precise (+/− 0.1 eV) calibration of the energy position of each individual energy scan. Repeated measurements were added after energy correction including spline fitting of smoothed data normalized to the primary X-ray intensity. Samples of reference compounds were measured in transmission mode. The mean intensity in the pre-edge region has been subtracted and the edge jump has been normalized to one by division by the mean intensity in the range 7200–7500 eV.

## Results

Following the histological procedures described in detail for homing pigeons [20], [32], we detected identical iron-mineral containing structures in the inner dermal lining of the upper beak (Figure 1) of the four avian species: homing pigeons (Figure 1A), garden warblers (Figure 1B), European robins (Figure 1C), and domestic chickens (Figure 1D). These structures exhibit the same light microscopical morphology and structural characteristics that we have previously seen in the upper beak of homing pigeons:

- Several dendrites in the stratum laxum of the dermis are filled with Prussian-Blue (PB) reactive material histochemically indicating a distinct local iron (Fe^3+^) concentration. In the newly investigated bird species, garden warbler, European robin and domestic chicken, the PB positive dendrites occur in several distinct areas near the lateral margin of the beak resembling the known pigeon micro-architecture.
- Over a length of 20 to 30 µm, the terminal regions of each dendrite (schematic drawing Figure 1E) contains 10–15 iron rich bullets (diameter about 1 µm) and one clear-cut vesicle (diameter about 5 µm, arrows in Figures 1A–D), which seems to be covered by an iron crust. Parallel dendrites may lie so close together that they appear as a common iron rich assembly containing about 20 bullets or more and two to three vesicles (Figure 1C, D, F).
- In all four bird species these dendrites usually lie regularly aligned along axon bundles (Figure 1F). Especially the dendrites in the caudal field are stretched strictly in a caudal to frontal direction.
- The dendritic assemblies do not build a dense chain but occur at a distance of about twice the length of the respective dendrites, when positioned on the same axon bundle (see arrow heads in Figure 1F, and Figure 2B inset, where several parallel bundles are obliquely cut).
  This regular pattern (neighboring dendrites close together and in parallel, but longitudinally at a regular distance) is an important tool to discriminate random iron concentrations from dendrites. Correlated with the site of marked dendrites in the PB-stained control sections (Figure 2A, B), selected tissue areas of all four bird species have been scanned for a topographic element analysis by microscopic synchrotron X-Ray fluorescence analysis (micro-SXRF).
- The dendritic site - as identified from the parallel control sections - was clearly discernable without additional histological staining procedures: (1) ‘dendritic’ iron follows a characteristic alignment as observed in the PB stained sections; (2) in all species, the dendritic site shows more than one magnitude higher Fe concentration compared to its direct vicinity (Figure 2C, Figure 3) (3) depending on the geometry, size and position of the axons within the respective section, Ca accumulation, which is a characteristic feature of nervous tissue, may often occur at relatively high concentrations next to Fe.
- Iron concentrations at several other locations outside the presumed sensory dendrite may be caused (1) by scattered or clotted erythrocytes, which may have accidentally remained inside blood capillaries in spite of the perfusion fixation; (2) by glandular tissue, which may accumulate iron depending on its physiological state; (3) by melanin, which can often be found in the stratum corneum of the cuticle surrounding the beak. Characteristically these melanin containing tissue strands (Figure 3) have a higher amount of copper (Cu) and a relatively lower content of iron than the dendritic sites [39]; (4) by accidental contamination, which can never be excluded (Figure 2C: point 1+3). The light microscopic view of the PB-marked iron containing dendrites in combination with the XRF-maps can be used to clearly discern dendrites from contamination.

Based on these findings, positions for micro-XANES analysis were selected from element maps around iron containing dendrites in the beak tissue of all four avian species (Figure 3, Figure 4, Figure 5). The micro-XANES measurements at every location were always repeated several times due to the generally low amount of iron present. No significant changes of the edge position or white line intensity were observed within a series of repeated scans; radiation damage or photo reduction is not considered as a critical issue under the present conditions. Repeated scans were added up for further analysis.

Also, the variation of spectra recorded at different potentially dendritic sites of each species is remarkably small. Figure 5 shows micro-XANES spectra recorded at nine different positions of a garden warbler sample (see Figure 2C). Based on the information from histology on parallel sections and micro-XRF element maps point 1 was identified as contamination, point 3 to 8 was assigned as potential dendrite positions and the allocation of point 2 and 9 was undefined. The XANES spectra however are separated in two groups: point 4 to 9 are very similar and are designated as dendrite type, point 1 and point 3 show a large metallic component and are probably due to contamination. The spectra recorded at point 2 are slightly shifted to lower energies compared to the dendrite-typical spectra. Small deviations between the XANES-data from different dendritic samples are expected, when the nervous endings were not completely contained inside the test specimen. Referring to the pigeon analyses [19], [20], we assume that the subcellular dendritic compartments in the bird species studied here, may also be composed of different iron minerals. Therefore, when the test sections only contain part of the dendrite, e.g. the vesicle and its next surrounding, the absorption spectra might be different from measurements of sections without the vesicle but containing several bullets.

For all bird species, the spectra of the dendritic sites were summed up and compared in Fig. 6. Again, the similarity of the avian spectra is striking, even if the total number of analyzed dendritic sites was not identical in all sections. Thus, the dendrites found in the skin of the upper beak of the four bird species seem to have the same iron mineral composition.

**Figure 7:**
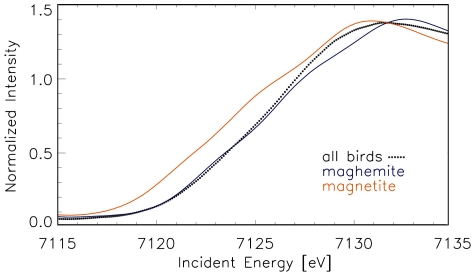
Summed micro-XANES spectra of biological (avian) tissue at dendrite positions compared to measured standard Fe compounds, magnetite and maghemite. The spectrum of the dendritic bird tissue does not completely match with the spectra of any Fe oxides. The maghemite spectrum (blue line) approaches the birds' spectrum (stippled black line) in the lower energy range very closely, but shows a significant deviation at energy above 7126 eV. However, the edge of magnetite (orange line) is shifted to lower energies, indicating its different oxidation state. Hence, the iron inside the biological material cannot be solely composed of magnetite.

A summation of all bird XANES spectra recorded at dendrite positions was compared to a collection of reference Fe oxide compounds, including the strongly ferromagnetic magnetite and maghemite (Figure 7). None of the reference materials has matched the material of the avian dendrites completely. The micro-XANES edge position of the iron containing dendrites spectra, which indicates the oxidation state of minerals in the dendrite (Figure 7 stippled black line), hints towards iron-III-oxides. The edge of the dendritic XANES spectra is significantly shifted to a higher energy compared to the edge of an iron-(II/III)-oxide. The spectrum of magnetite (Figure 7 yellow line) is clearly shifted to lower energies, according to its oxidation state as iron-(II/III)-oxide. The spectrum of maghemite, an iron-III-oxide, fits better (Figure 7 blue line), especially in the lower part of the edge. However the maghemite spectrum is not identical to the avian spectrum and deviates towards higher energies close to the white line maximum.

This finding suggests that the dendritic structures of the four bird species are similar. They contain mainly iron-III-oxides; a (minority) contribution of an iron-II/III-oxide cannot be excluded. However, the iron compound measured at the dendrite position cannot be composed of magnetite alone.

## Discussion

In the upper beak of garden warblers, domestic chickens, and European robins, we have found iron containing dendrites which closely resemble those described previously in homing pigeons [19], [32]. According to our histological data on these four different bird species, the dendritic system seems to have developed early in avian phylogeny and very little evolutionary change seems to have affected the basic dendritic structure since the radiation of birds, i.e. ‘after the split of the Galloanseres in the Mid-Cretaceous period’ [40] into the many taxa known today. This finding suggests that the iron containing dendritic system in the upper beak is a general avian feature important for their sensing of the Earth's magnetic field.

### A Common Structural Basis for the Putative Avian Magnetic Field Receptor

Despite of the so far restricted number of investigated avian species, we could not find hints toward principal species-specific differences in the histological characteristics of the iron containing dendrites in the inner dermal lining of the upper beak. The dendrites in garden warblers, European robins and domestic chicken seem to share the structural features previously described in pigeons, which suggest that they might be component parts of an avian magnetometer: (1) the dendrites occur in distinct dermal layers located near the rim of the upper beak, and clearly not as randomly distributed ‘waste packages’ of the iron-metabolism. (2) According to tracing and lesion experiments reported in different papers [41]–[43] the iron containing dendrites located in these beak areas are part of the sensory input system of the median branch of the ophthalmic nerve (ROM). According to our studies, the iron particles described in the ethmoid region in other animals (for example cribriform plate in fish [12]) may belong to the most caudal area of this dendritic system. (3) All the “naked” dendrites have the same or at least a very similar spindle shape, a length of about 20 to maximally 40 µm, and a diameter of about 5 µm. We could not detect any additional stimulus conducting systems like for example known from the corpuscular dermal receptor systems. This finding supports our interpretation that the structures are located directly inside nervous endings (for double staining of the dendrites with anti-neurofilament antibodies see [32]). (4) Each dendrite contains loosely arranged iron-rich bullets (diameter uniformly 1 µm) and a centrally located iron-coated vesicle (diameter 5 µm). These intracellular compartments are assumed to play an important role as a stimulus conducting system of magnetic field reception via these structures [19], [20]. This finding contradicts the assumption of an intracellular magnetic rod as key feature of a magnetoreceptor [31]. (5) Similar structural details (iron rich bullets and vesicles) cannot be detected in any other dermal cells or sensory organs, and never in the lower beak. This finding shows that the iron-containing compartments are special features of distinct terminals of the ROM. (6) Several dendrites seem to lie in parallel. Longitudinally, another dendritic group only can be found at a distance of about twice the length of a dendrite. This observation is to be expected if the dendrites behave like magnetic dipoles [34]. (7) This micromagnetic arrangement matches our previous magnetoreceptor hypothesis, which we now suggest to be valid for most if not all birds: soft magnetic material within explicit subcellular compartments enables a gradual sensory reaction with the changing intensity and/or direction of the magnetic field [19], [20].

Depending on the expected technical progress which could resolve iron concentrations in thicker sections or even whole mounts where the micro architecture of the iron containing dendrites is preserved and not disturbed by any sectioning process, we will be able to analyze the relative orientation of the dendritic system in greater detail [44]. A precise measurement of the natural variation of the dendritic alignment and bilateral symmetry will be essential for an estimation of the physiological range and dynamics of the sensory system.

### Different Magnetic Material May be Contained in the Avian Magnetoreceptor Candidate

Our model of an avian magnetometer [19], [20] depends on well-defined features of the iron minerals involved in these dendrites: a combination of soft and hard magnetic materials. In order to preserve the natural microstructure of the components during the physicochemical characterization, we used a brilliant, highly focused topographic analysis by means of microscopic X-ray absorption analyses in the near edge region. Micro-XANES is acknowledged as a reliable method to describe qualitatively (and nearly quantitatively) physicochemical characteristics of minerals and “mixtures” of various compounds. When accurately adjusted, the relative position and amplitude of pre-edge structure, edge position (for Fe around 7112 eV) and near-edge structures of XANES spectra (see Figure 7) of well-defined reference samples can be compared to spectra of an unknown composite [38]. These data can help to identify the iron oxides in the sensory dendrites, even when the minerals occur only in small amounts or have a poor crystallinity. The ‘edge’, the slope of the spectrum after the pre-edge peak, shifts to higher energy with increased oxidation state of the iron mineral. This helps to distinguish e.g., between magnetite (Fe (II)Fe(III)_2_O_4_) and maghemite (Fe(III)_2_O_3_), as magnetite contains both, iron(II) and iron(III), while maghemite, a ferrimagnetic mineral with similar magnetic properties as magnetite, is a pure iron(III)-oxide: The edge of magnetite is 1.6 eV lower than that of maghemite or other iron-III-oxides under study [38]. This result clearly shows that the dendrites in the avian beak mainly contain iron(III)-oxide and not pure magnetite, as predicted in several previous papers, where magnetite was assumed to be the only magnetic material underlying biological magnetoreception [24], [31], [45], [46].

Based on their diffraction pattern in the electron microscope, the little bullets inside the dendrites were assumed to be composed of nano-crystals of magnetite [47]. However, this method would be suitable for discrimination between magnetite and maghemite, only if the minerals would occur in larger amounts, since the diffraction patterns of both minerals are nearly identical and differ only in small details [48], which can be resolved only for larger sample masses. The XANES spectra of the dendrites suggest that magnetite is not the main constituent. Thus, care should be taken before using the well established notion of ‘magnetite-based magnetoreception’. Rather the term ‘iron mineral-based magnetoreception’ should be used - at least in birds.

The question which iron (III)-oxide(s) are contained in the dendrites is best evaluated by identifying the respective magnetic and crystalline properties of the minerals. In previous papers on the pigeon dendrites [35] based on a smaller number of samples, we have predicted that maghemite may account for most of the iron inside the dendrites, as the XANES spectra of maghemite reference samples approximate the spectra of the biological specimens. This view is shared by Tian et al. [49], who performed various physical tests, especially SQUID measurements of isolated beak skin samples and interpreted their data on the magnetic remanence as strong evidence that additionally to magnetite a second magnetic material - they propose maghemite - must be contained in the dendritic system.

Erroneously iron deposits, mainly biogenic magnetite, in the central nervous system (e.g. hippocampus [50]; in Alzheimer patients [51]; general overview: [52]) and mouth parts of invertebrates (e.g. radula of snails [53]; mandibles of arthropods [54]) have been discussed as putative sites of magnetoreception [24]. But randomly distributed iron deposits in the brain caused for example by inflammatory or degenerative processes cannot serve as structural basis for a receptor process occurring in healthy organisms. In “dead” body appendages metals may harden the delicate tips. These structures are clearly distinct from e.g., hairs which serve as stimulus conducting devices and always have a direct connection to sensory cells.

Up to date, besides the dendritic system in the avian beak, no structural candidates for a magnetic map sense have been described, which offer a site for the transduction of magnetic field parameters into a receptor potential and a sound concept for how the magnetic vector is detected.

### Iron-Based Magnetoreception as a Common Avian Characteristic

We interpret the structural and biophysical features of the iron containing dendritic system we found in all the four remotely related avian species we tested as supporting evidence for our hypothesis that these structures are the stimulus conducting device of a magnetoreceptor [19]: The little bullets, which are attached to the cell membrane, presumably strain sensitive membrane channels, which may be the site of the magneto-mechanical transduction process. The other intracellular iron containing components, platelet chains together with the vesicle, might provide a local amplification of the magnetic flux at the site of the bullets. This local amplification inside a dendrite depends on its alignment with the external field, thus inducing a strictly direction sensitive excitation, which has been modeled by mathematical simulations [36]. They have predicted a gradual membrane strain close to the magnitude described for mechanosensation. Thus, the avian ROM dendritic system is an appropriate candidate structure for a biological magnetometer. Additionally, the micro architecture of the ROM dendritic system, a three-dimensional alignment of the dendrites, strongly suggests a directional sensitivity of each dendrite and consequently a spatial characteristic of the dendritic entity.

The functional meaning of the ROM dendritic system as a map sense has been indicated by a few experiments, only (reviewed in [55]): (1) Strong magnetic pulses affected the orientation of birds for several days and evoked distinct deviations from the homing direction depending on the position of the bird inside the solenoid. (2) Anesthesia of the rim of the beak and (3) partial lesions of the trigeminal nervous pathways to the brain have delivered first evidence supporting a putative role of this system as a 3D-magnetometer. (4) Processing of magnetic field information via the trigeminal nerve could be demonstrated by electrophysiological recordings of direction sensitive action potentials [26]. More often the magnetic compass and not the magnetic map sense is topic of ongoing research. For example, recent data suggesting that the ophthalmic nerve is not necessary for magnetic compass orientation in orientation cages during migration, did not study the magnetic map sense, and explicitly stated that the presented data do not exclude a role of the ROM dendritic system in magnetic sensing [56].

The finding of similar iron-mineral based structures in three additional bird species from three orders (Galliformes, Columbiformes, Passeriformes) may at a first glance appear surprising since the orientation and navigation behavior of the bird species under study are fundamentally different: European robins are short to middle-distance migrants, and garden warblers are true long-distance migratory birds, while pigeons show reliable homing behavior typically in an area of about 100–200 km around their home loft. Even chicken, though resident birds use magnetic field parameters to find for example their mother [57]. However, all birds need to find their way over some distance and a magnetic compass and/or a magnetic map in particular [58] could be very useful at all navigation scales - provided that such a sense would be sensitive to minuscule changes in magnetic field intensity and inclination.
